# Development of a Novel Reference Material for Tumor Mutational Burden Measurement Based on CRISPR/Cas9 Technology

**DOI:** 10.3389/fonc.2022.845636

**Published:** 2022-04-28

**Authors:** Rongxue Peng, Guigao Lin, Lin Li, Jinming Li

**Affiliations:** National Center for Clinical Laboratories, Institute of Geriatric Medicine, Chinese Academy of Medical Sciences, Beijing Hospital/National Center of Gerontology, Beijing, China

**Keywords:** CRISPR/Cas9, tumor mutational burden, next-generation sequencing, reference material, standardization

## Abstract

As a biomarker that affects treatment decisions of immune checkpoint inhibitors, the accuracy, reliability, and comparability of tumor mutational burden (TMB) estimation is of paramount importance. To improve the consistency and reliability of these tests, qualified reference materials providing ground-truth data are crucial. In this study, we developed a set of formalin-fixed and paraffin-embedded (FFPE) samples with different TMB values as the novel reference materials for TMB estimation. By introducing several clinically relevant variants in MutS Homolog 2 (*MSH2*) gene and DNA polymerase epsilon (*POLE*) gene into human cell lines using CRISPR/Cas9 technology, we first constructed four typical cell lines which verified with hypermutator or ultramutator phenotype. Followed by cell mixing and paraffin embedding, the novel FFPE samples were prepared. It was confirmed that our novel FFPE samples have sufficient quantity of cells, high reproducibility, and they can provide matched wild type sample as the genetic background. The double-platform whole exome sequencing validation showed that our FFPE samples were also highly flexible as they containing different TMB values spanning a clinically relevant range (2.0–106.1 mut/Mb). Without limitations on production and TMB values, our novel FFPE samples based on CRISPR/Cas9 editing are suitable as candidate reference materials. From a practical point of view, these samples can be used for the validation, verification, internal quality control, and proficiency testing of TMB assessment.

## Introduction

Tumor mutational burden (TMB) is the total number of non-synonymous somatic mutations in the genomic coding area (1–4). In theory, the TMB level can reflect the probability of tumor neoantigen production and therefore, the likelihood of immune recognition and tumor cell killing (5). In recent years, numerous studies have confirmed that TMB, characterized by the number of somatic mutations derived from next-generation sequencing (NGS) approaches, can be used as a promising biomarker to predict the efficacy of immune checkpoint inhibitors (6–10). Hence, based on the abundant research data, guidelines from the European Society for Medical Oncology and the National Comprehensive Cancer Network have incorporated TMB as a biomarker in non-small cell lung cancer and gastric cancer in succession, recommending the combination of ipilimumab plus nivolumab or pembrolizumab as a first-line or second-line treatment for patients with high TMB (11–13).

As a biomarker that affects treatment decisions, the accuracy, reliability, and comparability of TMB estimation is of paramount importance (14–17). Currently, TMB is typically detected by whole exome sequencing (WES) and comprehensive genomic profiling. A series of U.S. Food and Drug Administration approved kits and laboratory-developed tests have been applied in clinical practice (18–20). However, as the testing panels, sequencing platforms, and bioinformatic algorithms differ widely across assays, and the mutation types considered for TMB estimation can vary from one laboratory to another, significant differences in TMB levels were always noticed between different assays and laboratories, especially when TMB values were around levels that may be clinical decision points (14, 15, 17, 21). Although many attempts have been made in recent years to improve the measurement of TMB, the inconsistency of testing is still an important problem that is yet to be resolved (14, 15, 17, 21). For clinical laboratories to improve the consistency and reliability of these tests, qualified reference materials that provide ground-truth data in harmonizing these measurements are of prime importance (21).

To date, the materials typically used are formalin-fixed and paraffin-embedded (FFPE) surgical specimens or paraffin-embedded human-derived tumor cell lines (with or without matched normal cell lines) (21, 22). However, owing to the difficulty of obtaining and mass-producing these materials, intratumor and intertumor heterogeneity (23, 24), and variations in the TMB levels in different patients (6, 25), FFPE samples from cancer patients display poor reproducibility. Meanwhile, since the validated TMB level in most human-derived tumor-normal matched cell lines that are publicly available and well-characterized is always lower than 10 mutations/megabase (mut/Mb) (22, 26), the applicability of tumor-normal cell line-derived samples is hampered by the limited number of cell lines with high TMB values and the cell types. For FFPE within tumor-only cell line samples, in spite of the diverse TMB values and cell types, they are not suitable for all NGS assays because they cannot provide a consistent genomic background. As such, it is obvious that these materials are not perfect as a standard for stringently assessing the performance of TMB estimation. To address these problems, a novel reference material should be developed. Since there is a high degree of variation in TMB levels across different cancer types (25), the ideal reference materials should be widely available, highly reproducible, and flexible enough to generate a series of standards with different TMB levels in ways that are applicable to different situations.

As many studies have demonstrated that mutations in the exonuclease domain of mismatch repair (MMR) genes and DNA polymerase epsilon (*POLE*) gene, which take charge of DNA repair in high-fidelity DNA replication, can cause a hypermutator or ultramutator phenotype (27–29). Hence, in this study, we developed a CRISPR/Cas9 knock-in system to introduce several clinically relevant variants in the MutS Homolog 2 (*MSH2*) and *POLE* gene into human cell lines, and constructed a series of cell lines with hypermutator or ultramutator phenotype. Followed by mixing with wild type cells at a range of precise ratios and paraffin embedding, thus, we developed a series of novel FFPE reference materials for TMB analysis. Without limitations on TMB level and production, these samples have a sufficient quantity of cells with high reproducibility, and they can be applied to any existing TMB estimation assay, since they can provide matched wild type sample as a genetic background. Moreover, apart from the FFPE sample, our reference material can be further prepared into any sample type, including circulating tumor DNA.

## Materials and Method

### Cell Line and Culture Conditions

HEK293T/17 cell line was chosen in our experiments as it is easy to culture and transfect. The cell line was purchased from American Type Culture Collection (ATCC, Manassas, USA), and cultured in Dulbecco’s Modified Eagle Medium (DMEM, Thermo Fisher Scientific, Waltham, USA) supplemented with 10% fetal bovine serum (FBS, Thermo Fisher Scientific, Waltham, USA) at 37°C and 5% CO_2_.

### Generation of Novel Cell Lines Co-Existing With *MSH2* and *POLE* Variants

**Figure 1:**
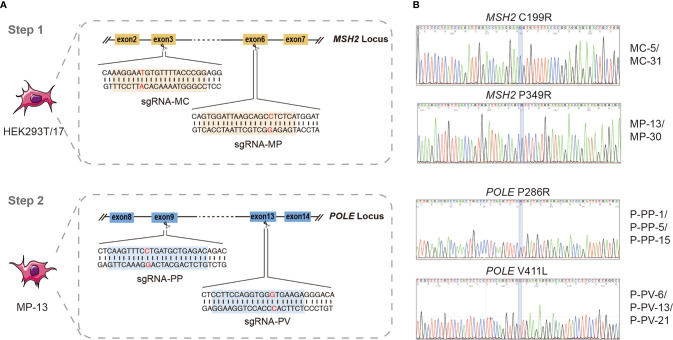
Generation of novel cell lines co-existing with *MSH2* and *POLE* variants by CRISPR/Cas9 system. **(A)** Schematic of the two-stage strategy for generating novel cell lines co-existing with *MSH2* and *POLE* variant. The sequence of sgRNAs for *MSH2* C199R (sgRNA-MC) and *MSH2* P349R (sgRNA-MP) are marked in yellow and sequence of sgRNAs for *POLE* P286R (sgRNA-PP) and *POLE* V411L (sgRNA-PV) are marked in blue. **(B)** Sequences of the PCR products showing the correct mutations of positive clones. 4 different *MSH2* ^mut^/*POLE* ^wt^ cell lines and 6 different *MSH2* ^mut^/*POLE* ^mut^ cell lines were ultimately constructed.

CRISPR/Cas9 gene editing technology was used to generate novel cell lines co-existing with *MSH2* and *POLE* variants in our study. The process of gene editing was fundamentally divided into two stages: cell lines containing *MSH2* variants was first generated by editing HEK293T/17 cells and then the cell lines containing the *MSH2* variant were further edited in order to generate cell lines co-existing with *MSH2* and *POLE* variants (**Figure 1**).

For each *MSH2* and *POLE* variant, single guide RNA (sgRNA) in close proximity to the site of mutation (less than 10 nt) was firstly designed *in silico* (http://crispor.tefor.net/; last access date: December 5, 2021). Subsequently, a suitable sgRNA was manually identified using the following criteria: a NGG motif on the 3′ end of sgRNA, a G (guanine) appended on the 5′ end of sgRNA, and a high on-target specificity and low off-target probability as possible (**Table 1**) (30, 31). DNA oligos containing the sgRNA sequences were synthesized and constructed into the Px458 vector (purchased from ATCC, Manassas, USA) expressing Cas9 endonuclease and green fluorescent protein.

**Table 1:** Sequences of sgRNAs and primers for each *MSH2* and *POLE* variant.

Target Site	sgRNA	ssODN	Forward primer to amplify target sites	Reverse primer to amplify target sites
*MSH2* C199R	5’-GCCGGGTAAAACACATTCCTT (AGG)-3’	5’-ATCTTGAGGCTCTCCTCATCCAGATTGGACCAAAGGAACGTGTTTTACCCGGAGGAGAGACTGCTGGAGACATGGGGAAACTGAGACAGA -3’	5’-TCAGTTTGAAGACATTCTCTTTGG -3’	5’-TCACTAGACTCAATTTGCTTACCTG -3’
*MSH2* P349R	5’- GTGGATTAAGCAGCCTCTCA (TGG)-3’	5’-AAAACCCCTCAAGGACAAAGACTTGTTAACCAGTGGATTAAGCAGCGTCTCATGGATAAGAACAGAATAGAGGAGAGATTGAATTTAGTG -3’	5’-TTTGGCTGGGGGAGAAATGT -3’	5’-GGTATAATCATGTGGGTAACTGC -3’
*POLE* P286R	5’-GTCTCAGCATCAGGAAACTTG (AGG)-3’	5’- ATCGATCATGTAGGAAATCATCATAATCTGGTCTGTCTCAGCATCACGAAACTTGAGGGGCAGTTTGGTCGTCTCAATGTCAAATGCCAA -3’	5’- GGGGAGTTTAGAGCTTGGCTT -3’	5’- TCCGTTCTTCCCACAATACCG -3’
*POLE* V411L	5’-GCCTTCCAGGTGGGTGAAGA (GGG)-3’	5’- AGAATCATCCTGGCTTCTGTTCTCATTCTCCTTCCAGGTGGTTGAAGAGGGACAGTTACCTTCCTGTGGGCAGTCATAATCTCAAGGCGG -3’	5’- GGTGCTTCCTTGTGTTGTGG -3’	5’- GTCTGAGGAGAGAACGCCAG -3’

Red “G”: for single guide RNA (sgRNA) not beginning with Guanine, an extra G was added to facilitate efficient transcription from U6 promoter; Green Base: altering variant.

For each transfection, well-dissociated cells at a density of 0.5×10^6^/mL were transfected using an electroporation kit (Celetrix LLC, Manassas, USA) on Celetrix LE+ machine according to the manufacturer’s instructions. 6 μg of sequence-verified plasmid (Px458 backbone) expressing specific sgRNA and Streptococcus pyogenes Cas9 (SpCas9) were transfected along with 3 μL of 100 μM single-stranded deoxyoligonucleotide (ssODN) (90 nt with 5′ PO_4_ and 3′ OH modification) (Sangon, Shanghai, China) carrying the mutation to be incorporated. After electroporation, the cells were plated onto 12-well plates with pre-warmed medium for further culturing. Simultaneously, SCR-7 (Selleck, Shanghai, China) was added at a final concentration of 1 μM for 48 h, with the aim of increasing the rate of homology directed repair (HDR). After 48 h, the transfection efficiency was assessed by observing the fluorescence intensity of green fluorescent protein, and then, the mixed clonal cells were digested for further investigation. A fraction of the cells was used to extract genomic DNA for evaluating the cleavage efficiencies of different sgRNAs by PCR and Sanger sequencing, whereas the remaining cells were flow cytometrically sorted with a SONY SH800 cell sorter (SONY Biotechnology, California, USA) into 96-well plates (Corning, Corning, NY) for monoclonal cell isolation and culture. Once the single-cell derived clones reached approximately 1×10^6^, the cells with CRISPR/Cas9-edited homozygous variants were rapidly screened by PCR and Sanger sequencing. The primers used for PCR are listed in **Table 1**.

### WES Validation for CRISPR/Cas9 Edited Cell Lines

Positive clones from the Sanger sequencing screen were subsequently detected by WES to ascertain the mutation spectra and TMB levels.

Briefly, genomic DNA was extracted using the QIAmp DNA Mini Kit (QIAGEN, Hilden, Germany) from the cell lines grown for 5–10 passages after editing. 400 ng extracted DNA was used for sequencing library preparation. By using a combination of Hieff NGS Ultima DNA Library Prep Kit for MGI (YEASEN, Shanghai, China) and 96rxn xGEN-lockdown-reagents (IDT, Coralville, USA), the sequencing libraries were prepared according to the manufacturer’s instructions. Sequencing was performed using 2 × 150 bp paired-end reads on DNBSEQ-T7RS (MGI, Shenzhen, China) with an average depth of 500× according to standard sequencing protocols. Data analysis was performed using a custom-processed pipeline. First, the raw reads were filtered to remove adapters and low-quality reads using fastp (version 0.20.1). Subsequently, alignment sorting, local realignment, duplicate removal, and base quality recalibration were performed using Sentieon (version 202010). Mutect2 (version 4.1.9) and SomVAS (an in-house software) were used to call and annotate the SNVs and indels.

The variant-calling criteria for TMB calculation were set as allelic depth ≥ 3, variant allele frequency (VAF) ≥ 5%, nonsynonymous mutations, and located in the coding regions. For the TMB calculation, the number of genomic alterations after the filtering process was divided by 32 Mb. R packages tidyverse, maftools, and deconstructSigs were used to analyze the mutation data.

### Preparation of FFPE Reference Materials

Confluent cell cultures were digested, and counted by Invitrogen Countess 3 FL (Thermo Fisher Scientific, Waltham, USA) prior to mixing and processing. Subsequently, according to the expected TMB level by calculating, cells with ultramutator phenotype were serially blended with the HEK293T/17 cells at precise ratios of 1:2, 1:3, 1:4, 1:5, 1:6, and 1:7, with total cells of 1×10^8^. The mixed cells were pelleted by centrifugation, and the medium was carefully aspirated. To fix cell pellets and construct a spatial network structure to agglomerate cells without affecting the cell shape, a cell block preparation kit (LBP, Guangzhou, China) was used. After 2 h, the cell pellet was carefully dislodged, wrapped in paper, and placed in processing cassettes. Dehydrating and paraffin embedding were subsequently automated performed using a Renaissance Tissue Processor (Ventana Medical Systems, Tucson, USA). Each paraffin-embedded cell block was cut into 10 μm-thick sections and used as the FFPE reference material for TMB analysis.

### WES Validation for FFPE Reference Materials

In order to estimate the TMB level of our novel FFPE reference materials, the genomic DNA extracted from 10 μm-thick FFPE slides using the GeneRead DNA FFPE Kit (QIAGEN, Hilden, Germany) was subjected to two reference laboratories for WES analysis (performed on DNBSEQ-T7RS [MGI, Shenzhen, China] and Novaseq 6000 [Illumina, Santiago, USA], respectively). Both assays have been strictly validated and have passed our multicenter assessment (unpublished data). The WES assay performed on DNBSEQ-T7RS was performed as described above, while the WES assay performed on Novaseq 6000 was processed as follows.

200ng DNA was sent for WES performed on Illumina Novaseq 6000 (2×150 bp) with libraries prepared using the Human Exome Library Preparation kit (Genetronhealth, Beijing, China) and sequenced to an average depth of 500×according to the manufacturer’s instructions. After removing adapters and low-quality reads by using FastQC (version 0.11.5) and Trimmoatic (version 0.36), the raw reads were aligned to the human genome (hg38) using BWA (version 0.7.17-r1194). Samtools (version 1.3), Picard (version 2.2.1), and GATK (version 3.5) were sequentially used for alignment sorting, duplicate removal, and base quality recalibration. Variant calling was performed using Mutect (version 3.1-0-g72492bb) and Strelka (version 2.9.2). An in-house software, Result_gather, was used to retain only high-confidence mutations for subsequent TMB estimation: VAF < 5%, allelic depth <7, and dbSNPs were all removed. For further TMB calculations, tmbTissue.py (in-house software) was used.

### Data and Statistical Analysis

The mutation data of lung adenocarcinoma (LUAD) were retrieved from The Cancer Genome Atlas database (https://portal.gdc.cancer.gov/legacy-archive) for mutational signatures analysis and comparison. WES data used for commutability assessment derived from our unpublished research and the Sequence Read Archive under accessions PRJNA307199 (32) and PRJNA293912 (33). Statistical analysis and data visualization were processed by R packages tidyverse, maftools, deconstructSigs, and GraphPad Prism 8.

## Results

### Generation of Novel Cell Lines Co-Existing With *MSH2* and *POLE* Variants Using the CRISPR/Cas9 System

In order to affect the cellular DNA repair function as well as generate cell line with hypermutator or ultramutator phenotype, two previously deemed Class 5 pathogenic *MSH2* variants (*MSH2* C199R and *MSH2* P349R) and two previously deemed Class 5 pathogenic *POLE* variants (*POLE* P286R and *POLE* V411L) were selected. A two-step CRISPR/Cas9 gene editing strategy, as outlined in **Figure 1**, was used to sequentially introduce each variant directly into the endogenous *MSH2* or *POLE* loci.

The first step of the genome editing process was to introduce variants directly into the endogenous *MSH2* locus of HEK293T/17 cells (**Figure 1A**). To do this, a plasmid vector-based expression of both sgRNA and SpCas9 was used to introduce a genomic DNA double-strand break at the appropriate location in *MSH2*. We then selected clones which successfully utilized homology directed repair (HDR) to site-specifically introduce the mutations in a homozygous fashion. To enhance the generation efficiency of single-cell derived clones carrying the desired mutation, SCR-7 (DNA ligase IV inhibitor) was used during the targeting process to reduce non-homologous end joining repair of DNA double-strand break and increase the HDR efficiency. As determined by Sanger sequencing screening, we identified homozygous clones with efficiencies ranging from 3.7–5.1%. Ultimately, four different *MSH2* homozygous variant-expressing cell lines were successfully generated in the first-round gene editing, including MC-5 and MC-31 for *MSH2* ^C199R/C199R^ cell lines, and MP-13 and MP-30 for *MSH2* ^P349R/P349R^ cell lines (**Figure 1B**).

The second step of the editing process began with the creation of the *MSH2* homozygous variant-expressing cell line (MP-13) described above (**Figure 1A**). A similar gene editing protocol was used to introduce variants directly into the endogenous *POLE* locus of MP-13. To select individual positive clones, we performed fluorescence-activated cell sorting, and eventually identified six novel cell lines co-existing with *MSH2* and *POLE* variants (P-PP-1, P-PP-5, and P-PP-15 for *MSH2* ^P349R/P349R^/*POLE* ^P286R/P286R^ cell lines; P-PV-6, P-PV-13, and P-PV-21 for *MSH2* ^P349R/P349R^/*POLE* ^V411L/V411L^) by sequencing the specific *MSH2* and *POLE* loci (**Figure 1B**). Higher editing efficiencies ranging from 14.3–15% were observed in secondary editing.

### Mutation Landscape and TMB Level of the Edited Cells

To ascertain the mutation landscape and TMB levels of each kind of edited cell, validation was performed for MC-5, MP-13, P-PP-1, and P-PV-6 as representatives. Using WES, which is the gold standard for TMB estimation, all samples were sequenced with an average coverage depth of more than 500×, and all target mutations had sufficient coverage to accurately detect variants with VAF as low as 5%. The wild-type HEK293T/17 cell line was also detected in parallel as a normal control to filter out irrelevant mutations and measure the TMB level of edited cell lines.

**Figure 2:**
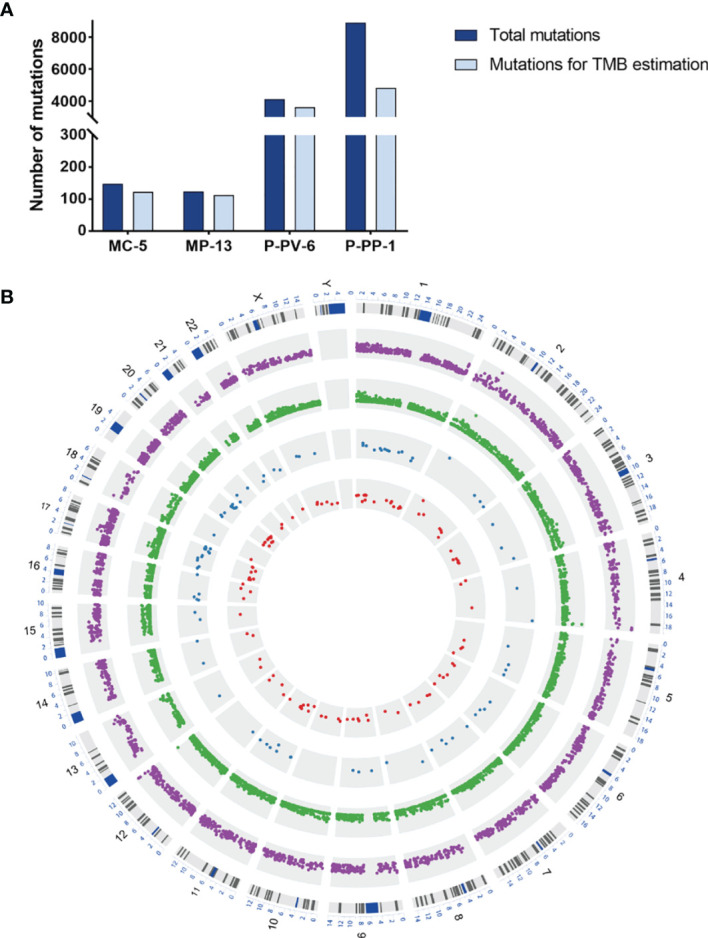
The mutation landscape and TMB levels of MC-5, MP-13, P-PP-1, and P-PV-6. **(A)** Distribution of total nascent mutations and nonsynonymous mutations in four engineered cell lines. **(B)** Circos Plot of detected nascent mutations in four engineered cell lines. Outer purple Track: P-PV-6. Middle green track: P-PP-1. Middle blue track: MP-13. Inner red track: MC-5.

Results obtained by WES revealed that, compared to the HEK293T/17 cell line, there was a clear increase of mutations in all these edited cell lines (**Figure 2A**). Of which, the number of detected somatic mutations in P-PP-1, and P-PV-6 (*MSH2* ^mut^/*POLE* ^mut^) was significantly higher than that in MC-5, MP-13 (*MSH2* ^mut^/*POLE* ^wt^). According to statistics, mutagenesis has respectively increased 1.22–1.25-fold and 8.31–16.8-fold in *MSH2* ^mut^/*POLE* ^wt^ cells and *MSH2* ^mut^/*POLE* ^mut^ cells over *MSH2* ^wt^/*POLE* ^wt^ control cells after editing. In all edited cells, the newly generated mutations randomly distributed in all chromosomes, both introns and exons, with VAF ranging from 2–99% (**Figure 2B**).

**Figure 3:**
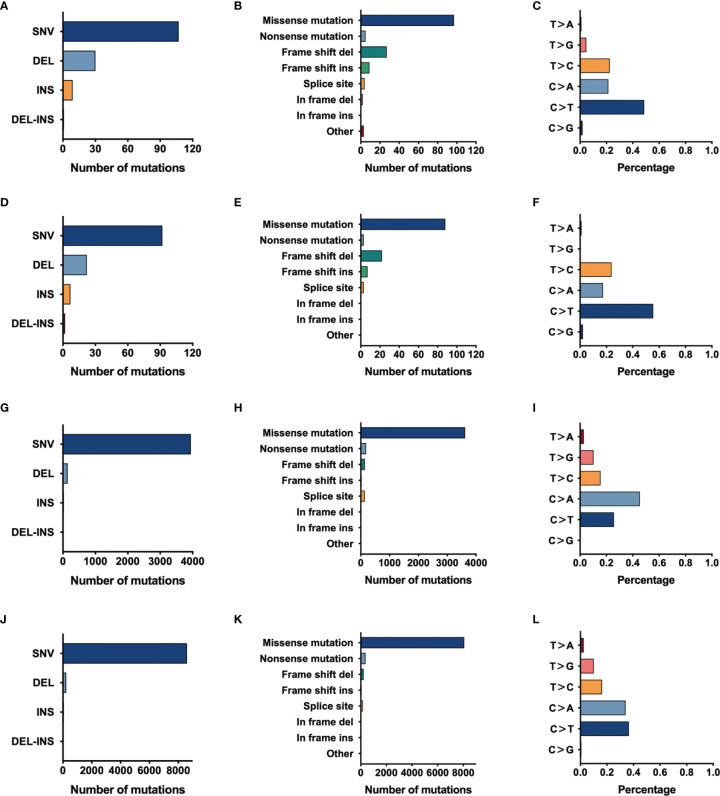
The mutational pattern of MC-5, MP-13, P-PP-1, and P-PV-6. **(A–C)** Total number of mutations detected by variant type, variant classification, and single-nucleotide variant (SNV) class in MC-5. **(D–F)** Total number of mutations detected by variant type, variant classification, and SNV class in MP-13. **(G–I)** Total number of mutations detected by variant type, variant classification, and SNV class in P-PP-1. **(J–L)** Total number of mutations detected by variant type, variant classification, and SNV class in P-PV-6.

**Figure 4:**
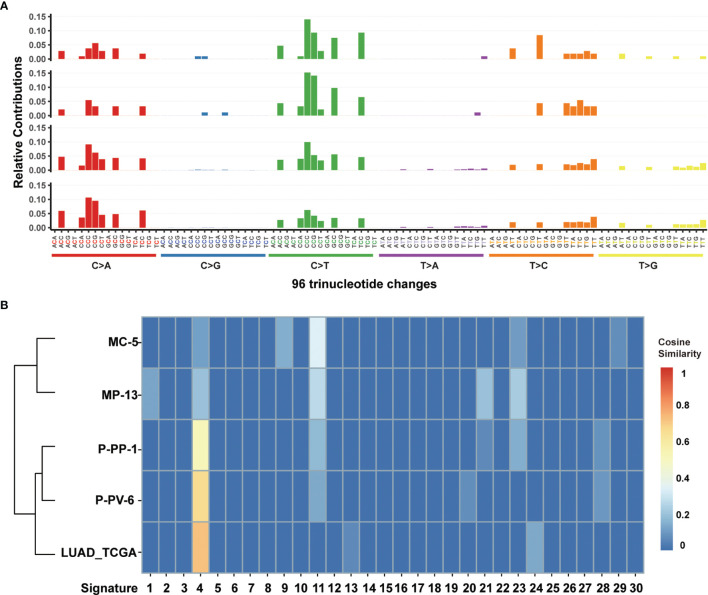
Mutational signature analysis of MC-5, MP-13, P-PP-1, and P-PV-6. **(A)** Mutational signature pattern of substitutions in MC-5, MP-13, P-PP-1, and P-PV-6 (the order of top-down) based on trinucleotide frequency. **(B)** Cosine similarity heatmap between the mutational profiles of MC-5, MP-13, P-PP-1, P-PV-6, and lung adenocarcinoma (LUAD) with high TMB.

For further exploring the differences between these engineered cell lines and authentic tumors, we then analyzed the mutational pattern and signature of nascent mutations in these four edited cell lines. It was found that, just as the mutational pattern of most tumors, the bulk of nascent mutations in our edited cells were SNVs (72.8–96.6%), with the remainder consisting of deletions and insertions (Indels, 2.5–20.4%) and Del-Ins (**Figure 3**). In MC-5 and MP-13 (*MSH2* ^mut^/*POLE* ^wt^), missense mutations were the most frequent alterations (65.99–71.54%), followed by frameshift Indels (23.58–24.49%), nonsense mutations (2.44–3.40%), and splice-site mutations (2.44–2.72%); whereas for P-PP-1 and P-PV-6 (*MSH2* ^mut^/*POLE* ^mut^), missense mutations mainly posed 87.84–90.58% of nascent somatic mutations (**Figure 3**). Almost half of this observed increase in P-PP-1 and P-PV-6 was due to mutations bearing strong similarity to those identified in tumors with concurrent DNA replication infidelity and MMR deficiency (34–36). These include C>A transversions and C>T transitions with a strong preference for NCC motifs. The signature of the remaining increased nascent mutations was also observed in MC-5 and MP-13, with a clear increase in C>T transversions in the CCG trinucleotide context, which is consistent with DNA damage arising from culturing cells *in vitro* (**Figure 4A**) (37, 38). Moreover, by analyzing the similarities of the mutational signatures, our edited cells also displayed features similar to those observed in LUAD with high TMB (**Figure 4B**).

By further filtering nonsynonymous mutations located in the coding regions, the calculated TMB levels for MC-5, MP-13, P-PP-1, and P-PV-6 were 3.8 (123 muts), 3.5 (113 muts), 113.3 (3626 muts), and 151.0 mut/Mb (4832 muts), respectively. It was found that although our edited cell lines will accumulate more mutations after several of passages, these nascent mutations during passages have little impact on TMB levels as most of the nascent mutations are located in non-coding regions. Take P-PP-1 as an example, although a total of 790 mutations have raised after 5 passages (from P5 to P10, approximately 15 generations), however, the calculated TMB levels climbed only 1.5 mut/Mb, as 113.3 mut/Mb (3626 muts) for P-PP-1 (P5) and 114.8 mut/Mb (3672 muts) for P-PP-1 (P10). Similarly, the calculated TMB levels of MP-13 raised from 2.6 mut/Mb (83 muts) to 3.5 mut/Mb (113 muts) after 8 passages (from P2 to P10, approximately 24 generations). Hence, it was demonstrated that these edited cells are feasible enough to prepare reference materials with specific TMB level.

### Characteristics and Validation Results of the FFPE Reference Materials

Based on genomic alterations characterized by WES, P-PP-1 was selected to further prepare FFPE reference materials with a gradient of TMB levels as it has an ultramutator phenotype. With a total of 1×10^8^ cells, P-PP-1 were serially blended with the HEK293T/17 cells at a range of precise ratios of 1:2, 1:3, 1:4, 1:5, 1:6, and 1:7 (named as P:H 1:2, P:H 1:3, P:H 1:4, P:H 1:5, P:H 1:6, P:H 1:7, respectively). After fixing, each cell pellet was embedded into small blocks as about 5–10 mm long, 5–10 mm wide, and 3–4 mm high. Besides, HEK293T/17 cells were also paraffin-embedded and applied as paired normal samples. To determine whether the amount of total DNA was sufficient to meet the requirements of subsequent detection, each FFPE cell block was sliced and subsequently extracted DNA. The total amount of genomic DNA in each section was higher than 1 μg, which was adequate for the detection of WES and comprehensive genomic profiling.

**Figure 5:**
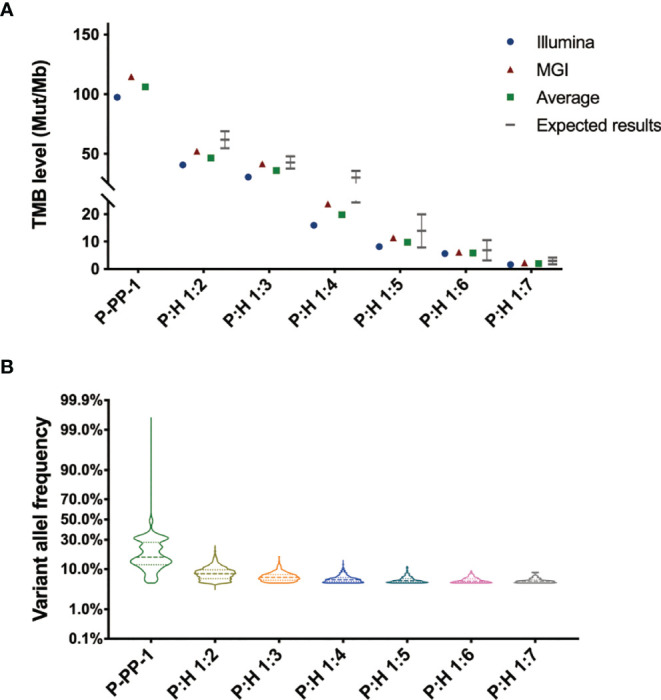
Sample validation of the novel FFPE reference materials by double-platform whole exome sequencing. **(A)** Sample validation by double-platform whole exome sequencing for the novel FFPE reference materials. Data showed in the form of mut/Mb. The mean values of all samples were consistent with their individual target values, spanning a clinically relevant range (2.0–106.1 mut/Mb). **(B)** Distribution of the variant allele frequency of nonsynonymous mutations in the novel FFPE reference materials.

In order to further identify the feasibility, homogeneity, and stability of our novel FFPE reference materials as well as determine the TMB level, a sample validation was carried out. Sections were randomly sampled from each type of FFPE block, and detected by two different WES assays performed on DNBSEQ-T7RS and Novaseq 6000 as previously described in the Methods section. With an average coverage depth of more than 500×, as well as sufficient coverage to accurately detect variants with VAF as low as 5%, the TMB values were obtained from each test, and the average of the two assays was used as the definite TMB level for the prepared reference materials. Depending on statistics, although the calculated TMB values differed slightly between different assays, the average results of P-PP-1 derived FFPE samples were basically in accordance with the TMB value detected by cells, as 106.1 ± 8.6 mut/Mb. The results of FFPE reference materials derived from gradient diluted P-PP-1 were also consistent with the theoretical results, spanning a clinically relevant range (2.0–46.1 mut/Mb), which partly reflects the homogeneity of our novel FFPE reference materials. The detailed results are shown in **Figure 5**. Besides, the stability assessment showed that no significant changes were observed in the amount of extracted DNA of our novel FFPE samples after storage at 4°C for 2 months. These results confirmed the feasibility, homogeneity, and stability of our novel FFPE reference materials.

Furthermore, in order to compare the commutability of our samples with the real clinical samples, we further compared the WES data of clinical samples which derived from previous published studies with those of our reference materials. The quality control statistics of the raw sequencing data showed that our FFPE samples-derived reads were similar to reads derived from clinical specimens in the sequencing quality pattern. The overall alignment rate of reads and average target capture efficiency of our novel FFPE samples were also approximating to those of the clinical specimens (overall alignment rate: 99.94% vs 99.96%; average target capture efficiency: 53.34% vs 60.64%; **Table 2**). Besides, we also investigated the mutational pattern of variants included in TMB calculation in our samples. The results showed that our samples contained different types of clinically relevant variations, ranging from SNVs to indels, just as the mutational pattern of clinical specimens. Hence, our novel FFPE samples based on CRISPR/Cas9 editing were confirmed with good commutability and availability as candidate reference materials for TMB estimation.

**Table 2:** Quality control statistics and mutational pattern comparison between the novel reference materials and real clinical samples.

Sample	Quality Parameters						Variant Type				TMB Value	
	Total reads	Reads mapped	Percent of reads on target	Mean targeted coverage(De-dup)	Target efficiency	Q30	SNV	del	ins	del-ins	Muts	Muts/Mb
P-PP-1	639790136	99.94%	57.50%	492.31	54.20%	96.14%	2866	84	15	0	2965	97.53
P:H 1:2	665295500	99.94%	55.90%	517.50	52.95%	96.24%	1226	10	2	0	1238	40.72
P:H 1:3	836595300	99.94%	58.01%	591.87	53.97%	96.34%	926	2	2	0	930	30.59
P:H 1:4	759738783	99.94%	56.05%	537.97	52.23%	96.53%	484	0	1	0	485	15.95
P:H 1:5	770632598	99.92%	54.50%	539.07	50.76%	95.83%	248	0	0	0	248	8.16
P:H 1:6	805576765	99.93%	58.18%	590.83	54.56%	96.08%	170	0	1	0	171	5.63
P:H 1:7	789148550	99.93%	58.49%	569.01	54.71%	96.12%	50	0	0	0	50	1.64
Patient 1	340621864	99.97%	55.93%	377.53	55.86%	97.67%	331	14	0	0	345	11.35
Patient 2	251488840	99.97%	68.65%	307.24	68.11%	97.40%	142	2	0	0	144	4.74
Patient 3	253431462	99.95%	60.26%	218.02	57.95%	97.59%	105	1	1	0	107	3.52

## Discussion

In the emerging clinical paradigm of ICI therapy, accurate detection is important to make the right decision for the treatment of cancer patients, for instance, in the case of TMB assessment for selecting the subgroup of patients receiving ipilimumab or pembrolizumab therapy (1). Among the numerous factors that affect the accuracy of TMB measurements, the lack of proper reference materials is particularly prominent (15, 17, 21). Therefore, to ensure the accuracy and reproducibility of TMB measurements, the present study sought to develop a kind of well-characterized candidate reference material.

As many studies have demonstrated that mutations affecting MMR and replicative DNA polymerases can cause rapid mutagenesis and a significant increase in TMB level (28, 34, 38), therefore in our study, we generated several cell lines with different TMB levels by introducing homozygous cancer-associated *MSH2* and *POLE* variants through the use of CRISPR/Cas9 system, and hereby developed a series of novel FFPE reference materials for TMB analysis by paraffin embedding these constructed cell lines. By using a two-stage gene editing process, four different *MSH2* ^mut^/*POLE* ^wt^ cell lines and six different *MSH2* ^mut^/*POLE* ^mut^ cell lines were finally constructed and named as MC-5, MC-31, MP-13, MP-30, P-PP-1, P-PP-5, P-PP-15, P-PV-6, P-PV-13, and P-PV-21, respectively (**Figure 1**). Of these, MC-5, MP-13, P-PP-1, and P-PV-6 were randomly selected as representative samples, and verified by WES to ascertain the mutation landscape and TMB levels of each kind of edited cell. Compared to the wild-type HEK293T/17 cells, a significant increase of mutations and TMB values was observed in these engineered cells, especially in *MSH2* ^mut^/*POLE* ^mut^ cells (P-PP-1 and P-PV-6, **Figure 2**). The mutational signature of these cells bore strong similarity to those identified in LUAD with high TMB, which confirms the availability of our edited cell lines (**Figures 3**, **4**). Besides, the relatively stable TMB level of these cells during passages also demonstrate the feasibility of the edited cell lines. After subsequent cell mixing, a series of cell pellets with different TMB estimates were obtained and embedded as FFPE blocks. With a sufficient amount of genomic DNA, our FFPE samples were detected by two different WES assays. The results indicated that our FFPE samples were homogenous and highly flexible as they contained different TMB values spanning a clinically relevant range (2.0–106.1 mut/Mb, **Figure 5**). Hence, we verified the suitability of our novel FFPE samples as reference materials for TMB assessment in clinical practice.

In contrast to the paraffin blocks of tumor tissues, our novel FFPE reference materials have proven to be sustainable and homogeneous. Since the cell lines can be grown *in vitro*, these edited cells and wild-type cells are potentially available in large quantities and can be mixed thoroughly, which makes this material easy to obtain and homogenize. In addition, unlike the FFPE within tumor-only cell line samples, our novel FFPE reference materials can generate paired variational and normal samples with the identical genetic background of HEK293T/17 cells. By sequence alignment with the genetic background, single nucleotide polymorphisms and germline mutations can be sufficiently filtered, and somatic mutations can be accurately identified by any existing NGS assays for TMB assessment. Not only that, our reference material panel can provide a series of control materials with precise TMB values spanning clinically relevant ranges, especially around levels that may be clinical decision points. Currently, many researches have shown that there is a high degree of variation in TMB levels across different cancer types, and their corresponding clinically applicable TMB threshold for therapy effectiveness of immune checkpoint inhibitors may also different (27, 39). Hence, the critical range controls (with TMB values ranging from 5 mut/Mb to 20 mut/Mb) are greatly needed for laboratories to validate and harmonize TMB-based assays. The edited cells bearing a hypermutation phenotype can be serially blended with the wild-type HEK293T/17 cells at a range of precise ratios to prepare panels in ways that are applicable for different cancers and situations. Furthermore, expect to provide reference materials for TMB values evaluation, our reference materials can also be used to evaluate the consistency and repeatability of somatic mutation detection since our serial FFPE samples were gradient diluted from the same cells. In other words, the laboratory can evaluate the detection consistency by comparing the detected mutations and their VAF in samples with different dilutions. For instance, mutations detected in P:H 1:7 samples should also be detected in P:H 1:6–P:H 1:2 samples, and with higher VAF values. Given all that, our reference materials can validate laboratory-developed tests and verify commercial detection kits. Meanwhile, since our standards are sustainable, homogeneous, and suitable for all existing TMB measurement, it is possible to perform internal quality control and proficiency testing of clinical laboratories that are using various assays by simply using our novel reference materials.

From a practical point of view, our edited cells can further be blended with other edited cells in the future, such as our previously edited cells containing the epidermal growth factor receptor gene (*EGFR*), *KRAS* proto-oncogene, GTPase gene (*KRAS*), and/or the echinoderm microtubule associated protein like 4 and ALK receptor tyrosine kinase (*EML4-ALK*) fusions (40–42). By this kind of mixture, we can simulate the authentic heterogeneous tumors. And as the mixed materials containing both numerous passenger mutations and driver mutations, they are applicable to validate the comprehensive genomic profiling tests as one-time used, thus avoiding repetitive and comprehensive validations. Apart from these, with the new cell lines generated by CRISPR/Cas9 system, circulating tumor DNA reference materials can also be generated by enzymatic digestion (43).

In this study, we have evaluated a type of novel FFPE sample as a candidate for reference materials for TMB assessment. The FFPE reference materials have been found to be widely available, highly reproducible, and flexible enough to generate a series of standards with different TMB levels in ways that are applicable for different situations.

## Data Availability Statement

The original contributions presented in the study are included in the article/supplementary material. Further inquiries can be directed to the corresponding author.

## Author Contributions

RP and JL contributed to conception and design of the study. RP, GL, and LL participated in the acquisition of data and implemented the analysis. RP and JL contributed to the writing of the manuscript. All authors contributed to the article and approved the submitted version.

## Funding

This study was supported by National Natural Science Foundation of China (No. 81902145; RP), and Beijing Municipal Natural Science Foundation (No. 7204299; RP). The funding sources have no role in the design and conduct of the study, collection, management, analysis, and interpretation of the data, preparation, review, or approval of the manuscript; and decision to submit the manuscript for publication.

## Conflict of Interest

The authors declare that the research was conducted in the absence of any commercial or financial relationships that could be construed as a potential conflict of interest.

## Publisher’s Note

All claims expressed in this article are solely those of the authors and do not necessarily represent those of their affiliated organizations, or those of the publisher, the editors and the reviewers. Any product that may be evaluated in this article, or claim that may be made by its manufacturer, is not guaranteed or endorsed by the publisher.

## Abbreviations

TMB, tumor mutational burden; FFPE, formalin-fixed and paraffin-embedded; WES, whole exome sequencing; NGS, next-generation sequencing; MMR, mismatch repair; sgRNA, single guide RNA; SpCas9, Streptococcus pyogenes Cas9; ssODN, single strand deoxyoligonucleotide; VAF, variant allele frequency; HDR, homology directed repair; LUAD, Lung adenocarcinoma.
